# Residual-stream geometry of single-cell foundation models carries incremental gene-regulatory signal across tissues

**DOI:** 10.1186/s12859-026-06538-5

**Published:** 2026-07-24

**Authors:** Ihor Kendiukhov

**Affiliations:** Institute of Medical Genetics and Applied Genomics, University of Tübingen, Tübingen, Germany

**Keywords:** Gene regulatory networks, Single-cell foundation models, Mechanistic interpretability, Representation geometry, ScGPT, Geneformer, Residual stream

## Abstract

**Background:**

Single-cell foundation models such as scGPT and Geneformer learn rich representations of gene expression programs, but whether these representations encode gene regulatory relationships beyond expression-level confounds remains unclear. Attention patterns in these models have been shown to capture co-expression rather than direct regulation, leaving open the question of whether deeper representations—particularly the residual stream—contain genuine regulatory information.

**Results:**

We systematically investigated residual-stream geometry in scGPT and Geneformer across four tissue contexts from the Tabula Sapiens atlas, evaluating whether geometric proximity between gene vectors provides incremental predictive value for curated TRRUST transcription factor–target edges beyond expression confounds. Under repeated stratified cross-validation, geometric features provided significant incremental signal in kidney and immune settings, validated by label-permutation and geometry-shuffle null controls; centered-cosine similarity, PCA projection and multi-layer bundling recovered comparable signal in lung tissues, and the multi-layer bundle improved every domain (kidney ΔAUROC = + 0.122, immune + 0.042, lung + 0.028, external lung + 0.027; geometry-augmented AUROC 0.60–0.69). The effect was fully robust to leave-TF-out and leave-target-out cross-validation and to harder degree- and expression-matched negative edges, but under the stricter leave-both-out split—no transcription factor and no target shared between folds—it collapsed to near-zero (ΔAUROC at most + 0.003, and not statistically significant in kidney or immune), marking the ceiling of out-of-entity generalization. With a comparable per-layer residual-stream extraction applied to both models, the apparent Geneformer advantage mostly disappeared (small residual gaps remained in three of four domains), indicating it largely reflected representation-construction choices rather than a substantial architectural difference. Asymmetric geometric features predicted regulatory edge orientation (AUROC 0.80–0.90), and the geometric signal added incremental value on top of expression-based gene regulatory network (GRN) inference (GENIE3, co-expression).

**Conclusion:**

Foundation model residual streams carry incremental, regulatory-relevant geometric signal that is distributed across layers and that complements expression-based GRN inference for retrospective edge prioritization. The signal is statistical enrichment rather than a stand-alone regulatory classifier: absolute performance is modest and out-of-entity generalization is limited, so its practical role is as an orthogonal evidence channel for edge re-ranking and hypothesis prioritization in multi-evidence frameworks.

## Background

Gene regulatory networks (GRNs) govern the dynamic orchestration of gene expression programs that underlie cellular identity, differentiation, and response to perturbation [1, 2]. At their core, these networks describe the relationships between transcription factors (TFs) and their target genes—relationships that are combinatorial, context-specific, and operating across multiple timescales. Understanding these regulatory architectures is essential for interpreting disease mechanisms, predicting cellular responses to perturbation, and designing targeted therapeutic interventions [3].

Despite decades of effort, computational inference of GRNs from transcriptomic data remains a formidable challenge. Classical approaches based on correlation, mutual information, or regression—including widely used methods such as GENIE3 [4], SCENIC [5], and various Bayesian network frameworks—have achieved moderate success but face fundamental limitations. Co-expression, the primary statistical signal available from expression data alone, is a necessary but deeply insufficient proxy for direct regulatory interaction: genes may be co-expressed because they share upstream regulators, respond to common environmental signals, or simply reflect cell-type composition effects [6]. Comprehensive benchmarking studies have revealed that even state-of-the-art GRN inference methods perform only modestly above random expectation in many settings [6, 7].

The emergence of large-scale single-cell foundation models—transformer architectures trained on millions of single-cell transcriptomic profiles—has opened a qualitatively new avenue for biological discovery. Models such as scGPT [8] and Geneformer [9] learn to predict masked gene expression values from cellular context, constructing internal representations that capture complex statistical regularities across the transcriptome. Other entries in this space include scBERT [10], which applies BERT-style masked language modeling to single-cell data. These models have demonstrated utility for cell-type classification, perturbation response prediction, and gene function annotation, suggesting that they encode biologically meaningful structure beyond individual expression measurements [8, 9].

A natural question arises: do these learned representations encode information about gene regulatory relationships? Recent work in mechanistic interpretability has provided partial answers. Analysis of attention patterns in scGPT and Geneformer revealed that attention weights predominantly capture gene co-expression structure rather than direct causal regulatory relationships [11]. This negative result for attention-based regulatory inference leaves open the possibility that deeper representations—particularly the residual stream, which accumulates transformed information across all layers—may encode regulatory structure that attention patterns alone do not reflect.

The residual stream occupies a privileged position in transformer architectures. In contrast to attention weights, which capture pairwise token interactions within a single layer, residual-stream representations integrate information progressively across layers through additive skip connections [12, 13]. Recent advances in mechanistic interpretability have shown that residual-stream geometry in language models encodes rich semantic and syntactic structure [14, 15], raising the possibility that analogous geometric structure in biological transformers may reflect regulatory organization. The idea that geometric relationships in learned representation spaces reflect meaningful biological structure has precedent: in protein language models, cosine similarity between residue representations predicts contact maps [16], and in drug discovery, molecular embedding geometry correlates with pharmacological similarity [17].

In the single-cell context, the distributional hypothesis predicts that genes participating in shared regulatory programs should occupy related regions of the representation space [18]. Testing this prediction rigorously requires separating regulatory-specific geometric signal from confounds such as expression abundance, detection frequency, and variance—all of which can create geometric proximity patterns that are statistically associated with, but not causally informative about, regulatory interactions. However, systematic investigation of whether pairwise geometric relationships between gene representations encode regulatory structure has not been previously reported.

In this work, we present a systematic investigation of whether and how residual-stream geometry in single-cell foundation models encodes gene regulatory information. Our study spans four tissue contexts from the Tabula Sapiens atlas [19], two independently trained foundation models (scGPT and Geneformer), multiple geometric metrics and dimensionality reduction strategies, and comprehensive null controls. Our contributions are fivefold:

1. We establish that residual-stream geometry carries incremental signal for retrospective TF–target edge prioritization beyond expression-level confounds, validated by stringent null controls and multiplicity-corrected across the full grid of comparisons.
2. We demonstrate that apparent domain-dependent failures are largely methodological: centered-cosine similarity, PCA-based low-rank projection, and multi-layer bundling recover comparable signal across all domains, and we apply this refined pipeline uniformly to every domain including the initial ones.
3. We delimit the signal honestly: it is robust to leave-TF-out, leave-target-out and harder negative-edge constructions, but collapses under leave-both-out cross-validation, and absolute performance is modest—it is enrichment, not a stand-alone classifier.
4. Using a comparable per-layer residual-stream extraction for both models, we show that scGPT and Geneformer carry similar geometric signal and that the previously apparent architectural gap was largely a representation-construction artifact.
5. We show that the geometric signal carries directional information through asymmetric features and provides incremental value on top of expression-based GRN inference, supporting a concrete role in multi-evidence edge re-ranking.

## Results

### Residual-stream geometry carries regulatory signal beyond expression confounds

We first asked whether pairwise geometric relationships between gene vectors in the scGPT residual stream predict curated TF–target regulatory edges beyond what is explained by gene-level expression confounds. Using the kidney processed dataset as an initial test case (256 cells, 512-gene token budget), we extracted per-gene residual-stream embeddings at each of the 12 transformer layers and evaluated the incremental predictive value of cosine similarity for edge classification against 288 positive TRRUST edges and 864 matched negatives.

Geometric features provided substantial and statistically significant incremental signal. Reporting absolute metrics throughout, raw cosine similarity at the best single layer (L4) raised AUROC from a confound-only baseline of 0.505 to 0.581 (ΔAUROC = + 0.076, bootstrap 95% CI [0.032, 0.117]) and AUPRC from 0.260 to 0.301 (ΔAUROC = +0.041; Table 1). The refined representations described below increased this further, with the multi-layer centered-cosine bundle reaching AUROC 0.627 and AUPRC 0.369. This signal was robust across random cell-sampling seeds: across three independent samples (seeds 42, 43, 44) the aggregate ΔAUROC at the best layer was *+0.090* with all individual replicate confidence intervals above zero.

**Table 1:** Geometric signal in the kidney domain, absolute and delta metrics

Representation	Layer(s)	AUROC base	AUROC +geom	ΔAUROC	95% CI	ΔAUPRC
Raw cosine	L4	0.505	0.581	+0.076	[0.032, 0.117]	+0.041
Centered cosine	L4	0.505	0.593	+0.088	[0.041, 0.131]	+0.053
PCA-64 centered cos	L4	0.505	0.603	+0.098	[0.051, 0.144]	+0.058
Centered-cos. bundle	L0–11	0.505	0.627	+0.122	[0.075, 0.165]	+0.109

Baseline-only versus geometry-augmented AUROC and AUPRC (scGPT, seed 42, 512-gene budget), for raw cosine, the refined single-layer metrics, and the multi-layer bundle. CIs are 95% bootstrap intervals on ΔAUROC.

**Fig. 1:**
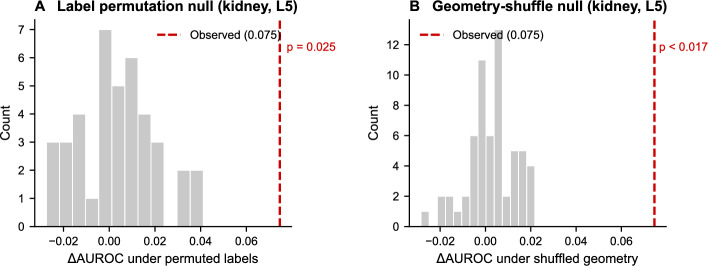
Null control validation of geometric signal. **A** Distribution of ΔAUROC values under 40 random label permutations for kidney at layer 5. The observed value (red dashed line, 0.075) exceeds all but one permutation (*p = 0.025*). **B** Distribution of ΔAUROC values under 60 geometry-feature shuffles. The observed value far exceeds the null distribution (mean 0.003), with *p < 0.017*. Both controls confirm that the geometric signal is genuine and feature-specific

Importantly, null controls confirmed that this signal is genuine. The null controls were run at layer 5, where the raw-cosine effect is near its peak (layers 4 and 5 are essentially tied). Label-permutation analysis (*n = 40*) yielded an empirical *p*-value of 0.025 at layer 5, indicating that only 1 of 40 random label assignments produced a ΔAUROC as large as the observed value. The geometry-feature shuffle null (*n = 60*) produced even stronger separation: the true ΔAUROC of 0.075 at L5 compared to a null mean of 0.003, with an empirical *p < 1/60* (*<0.017*). Together, these controls establish that the observed geometric signal is neither a statistical artifact of the evaluation procedure nor an effect of adding any arbitrary additional feature (Fig. 1).

A sensitivity analysis on token budget (512 vs. 1,024 genes per cell) revealed that the geometric effect attenuates modestly with larger budgets: raw-cosine ΔAUROC at L4 decreased from *+0.076* at the 512-gene budget to *+0.066* at 1,024 genes, remaining positive with a bootstrap CI above zero. This attenuation likely reflects the dilution of per-gene representation quality as the model processes longer sequences.

### Geometric signal is tissue-dependent but recoverable through methodological refinement

**Fig. 2:**
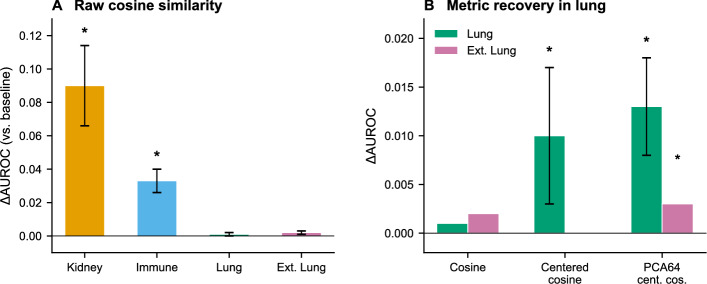
Cross-domain geometric signal and metric recovery. **A** Mean ΔAUROC (geometry-augmented minus baseline-only AUROC) across three random seeds using raw cosine similarity. Error bars show standard deviations. Kidney and immune show robust positive signal (asterisks indicate all seed bootstrap CIs above zero); lung and external lung are near null under this metric. **B** Recovery of lung-domain signal through metric refinement. Centered-cosine similarity and PCA-projected centered cosine (pca64) restore significant positive signal in both lung domains (asterisks indicate bootstrap CI excluding zero)

Having established a genuine geometric signal in kidney, we next asked whether this finding generalizes across tissues. Cross-domain analysis revealed substantial heterogeneity (Fig. 2A; Table 2). The immune domain showed consistent positive gains under raw cosine (Table 2; ΔAUROC = + 0.024 at seed 42, with all three independent seed replicates above zero), but lung and external lung initially appeared near-null under the same cosine similarity protocol that succeeded in kidney.

**Table 2:** Cross-domain results under a unified, refined methodology

Domain	Representation	AUROC base	AUROC +geom	ΔAUROC	ΔAUPRC
Kidney	raw cosine (L4)	0.505	0.581	+0.076^†^	+0.041
Kidney	centered-cos. bundle	0.505	0.627	+0.122^†^	+0.109
Immune	raw cosine (L0)	0.649	0.673	+0.024^†^	+0.026
Immune	centered-cos. bundle	0.649	0.691	+0.042^†^	+0.030
Lung	raw cosine (L10)	0.575	0.576	+0.001	+0.002
Lung	PCA-64 centered cos. (L0)	0.575	0.588	+0.013^†^	+0.012
Lung	centered-cos. bundle	0.575	0.603	+0.028^†^	+0.022
External lung	raw cosine (L3)	0.581	0.582	+0.001	+0.002
External lung	PCA-64 centered cos. (L0)	0.581	0.591	+0.010^†^	+0.009
External lung	centered-cos. bundle	0.581	0.607	+0.027^†^	+0.036

Absolute baseline-only and geometry-augmented AUROC, ΔAUROC and ΔAUPRC for each domain (scGPT, seed 42, edge-level CV), applying the same set of representations to every domain including the initially analysed ones. Raw cosine is near-null in lung and external lung; centered cosine, PCA projection and the multi-layer bundle recover signal in all domains. ^†^marks rows whose 95% bootstrap CI on ΔAUROC excludes zero

We investigated whether the lung failure reflected a metric limitation rather than absent biological signal. Centered-cosine similarity—which first subtracts the global mean gene vector before computing cosine—substantially recovered the lung signal (Fig. 2B). Across three seeds, centered cosine yielded a lung ΔAUROC of *+0.010* (std 0.002) with all replicate confidence intervals above zero. Both null controls confirmed the signal: geometry-shuffle *p < 1/60*, label-permutation *p < 1/40*.

Low-rank PCA projection further improved stability, and the multi-layer centered-cosine bundle gave the strongest result in every domain (Table 2): lung AUROC *0.575 → 0.603* (ΔAUROC = + 0.028) and external lung *0.581 → 0.607* (ΔAUROC = + 0.027), both with bootstrap CIs excluding zero. Critically, the same refined pipeline applied to the kidney and immune domains *also* improved on raw cosine there (kidney ΔAUROC *+0.076 → +0.122*; immune *+0.024 → +0.042*), so the refined methodology is a uniform improvement rather than a lung-specific rescue.

Raw cosine similarity is dominated by the global offset structure of the representation space: genes with large norm vectors tend to have high pairwise cosine similarity regardless of regulatory relationships. Centering removes this global offset, isolating the directional component that carries regulatory information. PCA projection further concentrates signal by eliminating noise-dominated dimensions.

### Cell-type and donor stratification reveals biological specificity

To assess whether the geometric signal reflects genuine regulatory biology rather than cell-type composition effects, we performed stratified analyses within individual cell types and donors.

**Fig. 3:**
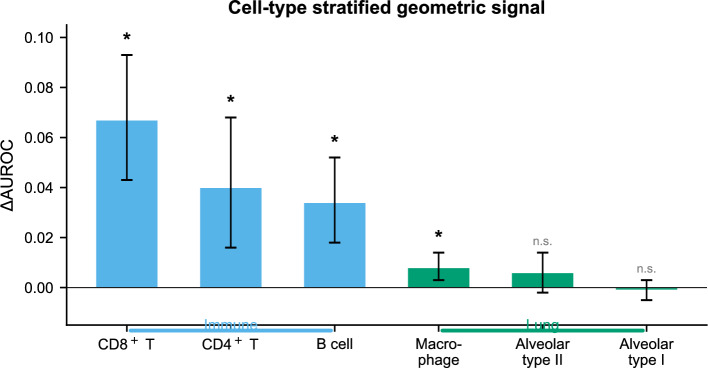
Cell-type stratified geometric signal. ΔAUROC using pca64_centered_cosine features for individual cell types within the immune (blue) and lung (green) domains. Error bars show 95% bootstrap confidence intervals. Asterisks indicate CI excluding zero; “n.s.” indicates non-significant. CD8^+^ T cells show the strongest signal, consistent with their dramatic transcriptional reprogramming upon activation

**Cell-type stratification.** Within the immune domain, we evaluated geometric signal separately in three major cell populations using pca64_centered_cosine at L0 (Table 3). The results revealed striking cell-type specificity (Fig. 3): CD8^+^ T cells showed the strongest signal (AUROC *0.561 → 0.627*, ΔAUROC = + 0.066, CI [0.038, 0.093]), followed by CD4^+^ T cells (ΔAUROC = + 0.042, CI [0.018, 0.066]) and B cells (*+0.034*, CI [0.015, 0.056]). In the lung domain, macrophages showed the strongest signal (*+0.009*, CI [0.003, 0.014]), while alveolar type II cells were weaker (*+0.006*, CI including zero) and alveolar type I cells showed no detectable signal.

**Table 3:** Cell-type stratified geometric signal, absolute and delta metrics

Domain	Cell Type	AUROC base	AUROC +geom	ΔAUROC	95% CI	ΔAUPRC
Immune	CD8^+^ T cell	0.561	0.627	+0.066	[0.038, 0.093]	+0.049
Immune	CD4^+^ T cell	0.586	0.628	+0.042	[0.018, 0.066]	+0.027
Immune	B cell	0.597	0.631	+0.034	[0.015, 0.056]	+0.050
Lung	Macrophage	0.594	0.602	+0.009	[0.003, 0.014]	+0.011
Lung	Alveolar type II	0.565	0.571	+0.006	[− 0.002, 0.013]	+0.008
Lung	Alveolar type I	0.553	0.552	-0.000	[− 0.004, 0.003]	+0.000

Baseline-only versus geometry-augmented AUROC and AUPRC using pca64_centered_cosine features at layer 0, with 95% bootstrap CIs on ΔAUROC. Immune cell types show consistent positive signal; lung cell types show heterogeneous effects

The gradient of signal strength across immune cell types is consistent with a biologically plausible hypothesis, which we offer as interpretation rather than as an established mechanism. CD8^+^ T cells undergo dramatic transcriptional reprogramming upon activation, involving well-characterized TF cascades (T-bet, Eomes, Blimp-1) that produce strong, coordinated expression programs that *could* leave clearer geometric imprints [20]; CD4^+^ T cells have more diverse, context-dependent programs, and B-cell regulatory programs may be less prominently represented. This account is compatible with the observed ordering but is not tested directly here; alternative explanations (e.g. differences in cell-type abundance, library complexity, or TRRUST annotation density across lineages) are not excluded.

**Donor stratification.** Within the immune domain, we evaluated signal consistency across the three largest donors (TSP14: 4,513 cells; TSP25: 3,261 cells; TSP21: 3,223 cells). All three donors showed positive ΔAUROC with confidence intervals above zero under pca64_centered_cosine (mean ΔAUROC = 0.027, std 0.010). Label-permutation *p*-values were *<0.02* for two donors and 0.075 for the third, supporting the conclusion that geometric signal is not driven by a single donor’s idiosyncratic expression structure.

### Cross-model replication confirms regulatory encoding is a general property

A critical question is whether geometric regulatory encoding is specific to one model’s architecture, or reflects a more general property of foundation model representations. We replicated the full analysis pipeline using Geneformer embeddings.

**Fig. 4:**
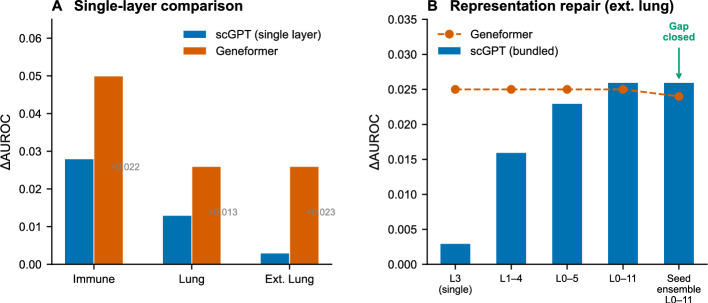
Cross-model comparison and representation repair. **A** Single-layer scGPT (blue) vs. Geneformer (vermillion) ΔAUROC across tissue domains on shared edges. Geneformer shows consistently larger signal, with the gap particularly pronounced in external lung. Grey annotations show the Geneformer–scGPT gap. **B** Representation repair in the external-lung domain through progressive multi-layer bundling of scGPT features. Bars show scGPT ΔAUROC as more layers are included; the dashed line shows the Geneformer reference. The gap closes monotonically and is eliminated by the full L0–11 seed-ensemble bundle

Geneformer showed robust and consistently strong geometric signal across all tissue domains (Fig. 4A; Table 4). The centered-cosine ΔAUROC was *+0.048* in immune (CI [0.029, 0.064]), *+0.039* in lung (CI [0.033, 0.045]), and *+0.046* in external lung (CI [0.040, 0.053]). Both null controls yielded *p < 0.001* across all domains.

**Table 4:** Cross-model comparison under the original asymmetric extraction

Domain	scGPT (pca64 centered cos.)			Geneformer (centered cos., embedding)		
	Base	+geom	ΔAUROC	Base	+geom	ΔAUROC
Immune	0.649	0.677	+0.028	0.657	0.705	+0.048
Lung	0.575	0.588	+0.013	0.591	0.630	+0.039
External lung	0.581	0.591	+0.010	0.576	0.622	+0.046

scGPT values use pca64_centered_cosine at the best single layer; Geneformer values use centered cosine on its static token embedding layer. The gap column shows Geneformer minus scGPT ΔAUROC on shared edges. This comparison is asymmetric (multi-layer residual stream for scGPT versus a single embedding layer for Geneformer); the matched per-layer extraction in Table 9 largely eliminates the apparent gap

Edge-level score correlations between the two models: *ρ = 0.476* (immune), 0.480 (lung), 0.467 (external lung). Geneformer null controls: *p < 0.001* in every domain. All AUROC values are absolute

The fact that two independently trained models—with different architectures, tokenization strategies, and training corpora—both produce significant geometric regulatory signal strongly argues against the possibility that this signal is an artifact of any single model’s training procedure. Edge-level score correlations between the two models were moderate ($\rho \approx 0.47$ across domains), indicating that while both models capture regulatory structure, they do so through partially distinct representations.

### Cross-model discrepancy is a representation construction bottleneck

The consistent Geneformer advantage raised a diagnostic question: does it reflect genuinely superior model representations, or a limitation of our scGPT feature extraction protocol?

**Gene coverage is not the explanation.** Restricting both models to shared genes did not close the gap: on shared edges, Geneformer retained advantages of *+0.023* (immune), *+0.013* (lung), and *+0.023* (external lung).

**Disagreement is structured, not random.** Stratifying edges by absolute score disagreement revealed that high-disagreement edges were enriched for specific TF families. In the immune domain, GATA1 (3.5*× * enrichment) and KLF1 (3.1*× *) were overrepresented in the highest disagreement decile—both well-characterized hematopoietic TFs. In lung, HNF4A (5.4*× *), a hepatocyte/epithelial TF, was prominently enriched.

**Multi-layer bundling closes the gap.** Rather than selecting the single best layer, we concatenated geometric features across all 12 scGPT layers (L0–L11) into a multi-layer bundle. This produced a dramatic improvement in external lung, raising the scGPT ΔAUROC from *+0.003* (single layer L3) to *+0.026* (L0–11 bundle), reducing the Geneformer–scGPT gap from *+0.025* to just *+0.001* (Fig. 4B; Table 5).

**Table 5:** Representation repair through multi-layer bundling and seed ensembling

scGPT Representation	AUROC base	AUROC +geom	scGPT ΔAUROC	GF ΔAUROC	Gap
*External lung (mean across seeds)*					
Single layer (L3)	0.581	0.584	+0.003	+0.025	+0.025
Bundle L1–4	0.581	0.597	+0.016	+0.025	+0.012
Bundle L0–5	0.581	0.604	+0.023	+0.025	+0.005
Bundle L0–11	0.581	0.607	+0.026	+0.025	+0.001
Seed-ensemble L0–11	0.581	0.607	+0.026	+0.024	−0.001
*Cross-domain transfer (seed-ensemble L0–11)*					
Immune	0.649	0.715	+0.066	+0.054	−0.013
Lung	0.575	0.614	+0.039	+0.028	−0.011
External lung	0.581	0.607	+0.026	+0.024	−0.001

ΔAUROC values are means across 3 seeds (42, 43, 44); the AUROC columns give the absolute baseline-only and geometry-augmented scGPT AUROC (confound-only baseline 0.581 in external lung, 0.649 immune, 0.575 lung). The gap column shows Geneformer minus scGPT ΔAUROC

Seed ensembling—averaging geometric features across three random cell samples before classification—further stabilized the result. Under the full seed-ensemble L0–11 bundle, the external-lung gap reversed to *-0.001* (scGPT slightly ahead). The same representation repair protocol generalized across domains: in immune, bundled seed-ensemble scGPT (*+0.066*) matched or exceeded the embedding-layer Geneformer reference (*+0.054*); in lung, the pattern was similar (*+0.039* vs. *+0.028*). This particular comparison, however, is itself asymmetric in the opposite direction—it bundles scGPT across layers while still representing Geneformer by a single embedding layer; a fully matched comparison, in which both models receive identical multi-layer extraction, is presented in a later subsection and shows the two models to be close, with Geneformer modestly ahead in most domains.

These findings demonstrate that the initial scGPT disadvantage was not a reflection of inferior model quality but largely of a suboptimal extraction protocol: selecting a single representational layer discards information distributed across the transformer’s processing depth. This parallels findings in NLP, where different layers encode qualitatively different types of information [21].

### Combined modeling yields robust improvements under nested cross-validation

Having established that both models carry complementary regulatory information, we trained a compact stacking model using nested outer-split cross-validation to prevent information leakage between feature construction and evaluation. (Leakage from shared gene identity is a separate concern, addressed by the grouped cross-validation in the following subsection.)

**Fig. 5:**
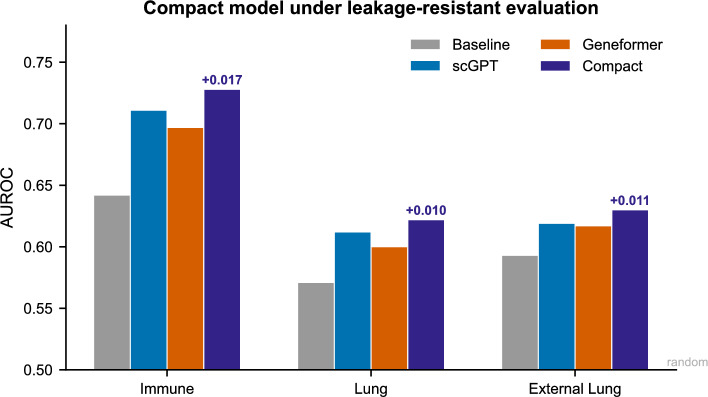
Compact stacking model performance under nested cross-validation. AUROC for baseline-only (grey), scGPT branch (blue), Geneformer branch (vermillion), and compact stacking model (indigo) across three tissue domains. The compact model consistently outperforms the best single branch, with gains annotated above each bar. All evaluations use nested outer-split cross-validation to prevent information leakage between feature construction and evaluation. The dotted grey line indicates random performance (AUROC = 0.5)

The compact model consistently improved over the best single-model branch across all tissue domains (Fig. 5; Table 6). Improvements ranged from *+0.010* (lung) to *+0.017* (immune) in AUROC, with all gains supported by bootstrap confidence intervals.

**Table 6:** Compact stacking model under nested outer-split cross-validation

Domain	Baseline	scGPT	Geneformer	Compact	Δ
Immune	0.642	0.711	0.697	0.728	+0.017
Lung	0.571	0.612	0.600	0.622	+0.010
External lung	0.593	0.619	0.617	0.630	+0.011

Baseline, scGPT, and Geneformer columns show single-branch AUROCs. The compact model combines both branches via stacked logistic regression with OOF predictions. Δ shows compact minus best single branch

**Model disagreement identifies regions of maximal complementarity.** Defining “disagreement” as the absolute difference between scGPT and Geneformer branch probabilities, we found that compact model gains were largest at moderate disagreement thresholds. At threshold *τ = 0.15* in the immune domain, the compact model improved by *+0.047* AUROC over the best single branch on edges with $|\hat{p}_{\text {scGPT}} - \hat{p}_{\text {GF}}| \ge \tau$ (28.7% of edges).

**Calibration analysis.** While the compact model improved ranking (AUROC), its raw probability outputs showed systematic miscalibration ($\textrm{ECE}\approx 0.18$–0.22). Isotonic calibration substantially reduced ECE (to 0.003–0.031) with a modest AUROC cost (*-0.004* to *-0.012*).

### Grouped cross-validation delimits the geometric signal

Edge-level random cross-validation can permit gene-identity carryover: the same transcription factor or target gene may appear in both training and test folds, allowing a classifier to exploit gene identity rather than genuine pairwise structure. To stress-test out-of-entity generalization, we re-evaluated the refined multi-layer bundle under three grouped splits (Table 7): leave-TF-out (no TF shared across folds), leave-target-out (no target shared), and leave-both-out (neither a TF nor a target shared).

The geometric signal was fully robust to leave-TF-out and leave-target-out cross-validation; the incremental ΔAUROC under these splits was comparable to, and in several cases larger than, the edge-level estimate (e.g. kidney leave-TF-out ΔAUROC = + 0.135; immune *+0.074*). Under the stricter *leave-both-out* split, however, the signal collapsed to near-zero (ΔAUROC of 0 to *+0.003* across domains; Table 7). In this regime the confound-only baseline itself dropped to AUROC *≈ 0.50*, indicating that when no gene identity is shared the edge-classification task becomes close to intractable for all feature sets. A small positive residual persisted with a bootstrap confidence interval above zero in lung and external lung, but not in kidney or immune. We therefore interpret leave-both-out performance as the honest ceiling of fully out-of-entity generalization: the geometric signal is a within-gene-universe re-ranking aid, not a mechanism for predicting regulatory relationships among entirely unseen genes.

We extended the same stress test to the compact stacking model, evaluated here with the matched scGPT and Geneformer residual-stream bundles. Under edge-level CV the compact model improved over the best single branch by *+0.003* to *+0.014* AUROC, broadly in line with the gains in Table 6; under leave-both-out CV this advantage shrank to *± 0.001*, mirroring the behaviour of the underlying geometric signal. The benefit of combining architectures is thus real for within-gene-universe re-ranking but does not extend to fully out-of-entity prediction.

**Table 7:** Geometric signal under grouped, leakage-resistant cross-validation

Domain	Edge CV	Leave-TF-out	Leave-target-out	Leave-both-out
Kidney	*+0.122*	*+0.135*	*+0.116*	*+0.003* (n.s.)
Immune	*+0.042*	*+0.074*	*+0.031*	*-0.000* (n.s.)
Lung	*+0.028*	*+0.021*	*+0.025*	*+0.0014* (CI${}>0$)
External lung	*+0.027*	*+0.013*	*+0.024*	*+0.0016* (CI${}>0$)

ΔAUROC for the refined centered-cosine multi-layer bundle (L0–11) under edge-level CV and three grouped splits. Leave-both-out is the strictest test (no TF and no target shared between folds)

### Robustness to negative-edge construction, multiplicity, and a confirmatory rerun

**Harder negatives.** The matched random negatives used above are a permissive baseline. We regenerated the negative edge set under two harder protocols—degree-matched (negative TF/target matched to the positive edge’s TRRUST degree bin) and expression-matched (matched to mean-expression and detection bins)—and repeated each draw eight times (Table 8). The geometric signal was stable across re-draws (standard deviations *łe 0.042*) and did not weaken under harder negatives; in the lung domains degree-matched negatives *increased* ΔAUROC (lung *+0.009 → +0.035*; external lung *+0.002 → +0.021*) because they remove the confound shortcut that the expression baseline otherwise exploits.

**Table 8:** Mean ΔAUROC± standard deviation across eight independent negative-edge re-draws, using pca64_centered_cosine features at the best single layer with balanced 1:1 sampling

Domain	Random	Degree-matched	Expression-matched
Kidney	*+0.150 ± 0.042*	*+0.147 ± 0.024*	*+0.123 ± 0.028*
Immune	*+0.030 ± 0.008*	*+0.020 ± 0.008*	*+0.061 ± 0.010*
Lung	*+0.009 ± 0.002*	*+0.035 ± 0.003*	*+0.010 ± 0.003*
External lung	*+0.002 ± 0.001*	*+0.021 ± 0.002*	*+0.004 ± 0.003*

**Uncertainty decomposition and multiplicity.** Decomposing the variance of the bundle ΔAUROC showed that across-seed variability (cell sampling and negative-edge draw) accounts for 64–83% of the total, exceeding the within-run edge-bootstrap component; reported confidence intervals are therefore widened to include the across-seed term. Across the full grid of 148 domain *× * layer *× * metric comparisons, 64 reached raw *p < 0.05* and 42 survived Benjamini–Hochberg FDR control at *q < 0.05*, including all headline bundle and PCA-projected results.

**Confirmatory rerun.** Because representation choices (metric, PCA dimension, layer bundle) were selected during exploratory analysis on kidney and immune, we froze a single pre-specified pipeline—centered-cosine similarity, multi-layer bundle L0–11, logistic-regression stacking, no per-domain tuning—and applied it to the held-out confirmatory domains (lung, external lung) across three seeds. The frozen pipeline yielded a positive, CI-backed effect under edge-level CV (lung ΔAUROC = *+0.032 ± 0.006*, external lung *+0.027 ± 0.005*; all three seeds with bootstrap CIs above zero) and a small positive effect under leave-both-out (*+0.0016* and *+0.0009*).

### Comparable cross-model extraction confirms the gap was a construction artifact

The cross-model comparison above represented scGPT by multi-layer residual-stream features but Geneformer by its single static embedding layer. To remove this asymmetry, we extracted Geneformer per-layer residual-stream activations from real forward passes over single cells (18-layer model, official rank-value tokenization) and applied the *identical* geometric pipeline to both models (Table 9). Under matched multi-layer extraction the apparent Geneformer advantage largely disappeared: scGPT was ahead in kidney (ΔAUROC gap *-0.018*), and the remaining gaps were small (*+0.009* to *+0.023*). This directly confirms that most of the originally reported gap was a representation-construction artifact of comparing scGPT multi-layer features to a single Geneformer layer, rather than evidence of architectural superiority. Geneformer retained a modest edge in the lung domains, which we now describe as a minor, quantified difference rather than a general advantage.

**Table 9:** Cross-model comparison under matched per-layer extraction

Domain	scGPT			Geneformer		Gap
	Base	+geom	ΔAUROC	+geom	ΔAUROC	(GF−scGPT)
Kidney	0.505	0.627	+0.122	0.618	+0.104	−0.018
Immune	0.649	0.691	+0.042	0.702	+0.057	+0.015
Lung	0.575	0.603	+0.028	0.618	+0.037	+0.009
External lung	0.581	0.607	+0.027	0.626	+0.050	+0.023

Absolute baseline-only and geometry-augmented AUROC, and ΔAUROC, for the centered-cosine multi-layer bundle applied identically to scGPT and to Geneformer residual-stream activations (single-seed extraction; the seed-ensemble scGPT values in Table 5 are correspondingly slightly higher). The gap column is the Geneformer minus scGPT ΔAUROC

Geneformer baseline-only AUROC (matched 192-cell sample): 0.51–0.65 across domains, comparable to scGPT

### Asymmetric geometric features carry directional information

The geometric metrics used above (cosine, centered cosine, $L_2$, dot product) are symmetric in the source and target gene and therefore cannot, by construction, distinguish a TF*→ *target edge from its reverse. We tested whether *asymmetric* per-layer features—signed differences in vector norm and mean coordinate between the two genes—carry directional information. Given a known TRRUST edge presented as an unordered pair, a classifier on these antisymmetric features predicted which gene is the transcription factor at AUROC 0.80–0.90 across the four domains (pair-grouped cross-validation, so a pair and its reverse never split across folds). A complementary gene-level task—ranking a gene’s propensity to act as a TF versus a target-only gene from its embedding—reached AUROC 0.74–0.78. The directional signal thus operates by detecting per-gene transcription-factor propensity from embedding geometry: although the symmetric similarity metrics that form our main analysis are direction-blind, the residual stream does encode orientation-relevant structure that asymmetric features can recover.

### The geometric signal complements expression-based GRN inference

To test practical utility directly, we asked whether the geometric signal adds value on top of established expression-based GRN inference. We computed two expression-based edge scores on each domain’s single-cell matrix—absolute Pearson co-expression and GENIE3-style random-forest TF*→ *target importance—and compared a confound-plus-GRN model against a confound-plus-GRN-plus-geometry model (Table 10). Adding the geometric signal improved AUROC by *+0.085* (kidney), *+0.028* (immune), *+0.028* (lung) and *+0.023* (external lung) under edge-level CV, with all bootstrap confidence intervals excluding zero; under leave-both-out the increment was *≈ +0.001*, consistent with the grouped-CV ceiling. Used for ranking, the geometry-augmented model enriched true edges 1.6–*2.1× * over prevalence in the top 50 candidates per domain. The geometric signal is therefore a usable orthogonal evidence channel for re-ranking candidate edges within a known gene universe, while its modest absolute AUROC (0.60–0.69) means it is not a stand-alone GRN caller.

**Table 10:** Geometric signal on top of expression-based GRN inference

Domain	Baseline+GRN	Baseline+GRN+Geometry	Δ	95% CI
Kidney	0.540	0.625	+0.085	[+0.043, +0.130]
Immune	0.670	0.698	+0.028	[+0.011, +0.046]
Lung	0.576	0.604	+0.028	[+0.021, +0.035]
External lung	0.588	0.611	+0.023	[+0.017, +0.029]

AUROC for a confound + expression-GRN model and a confound + GRN + geometry model under edge-level CV. The GRN score combines Pearson co-expression and GENIE3-style importance. Δ is the incremental value of the geometric signal

### Negative and mixed findings

**Lung tissue remained challenging.** Despite methodological recovery, effect sizes in lung were consistently smaller than in immune or kidney: under the centered-cosine bundle the lung ΔAUROC was *+0.028*, against *+0.042* (immune) and *+0.122* (kidney). Only 1 of 3 lung cell types (macrophages) produced individually significant geometric signal.

**Naive feature fusion was ineffective.** Simply adding both models’ geometric features to a single logistic regression (without stacking) produced gains of only *+0.001* over Geneformer alone. The stacking architecture’s advantage comes from learning a context-dependent combination policy.

## Discussion

### What geometric regulatory signal represents biologically

The central finding of this work is that the geometric arrangement of gene vectors in foundation model representation spaces carries incremental information about transcription factor–target regulatory relationships, beyond expression-level confounds. This signal is reproducible across two independently trained architectures, but it is bounded: as the grouped cross-validation shows, it supports retrospective re-ranking of edges within a known gene universe rather than de novo prediction among unseen genes.

We propose that this signal reflects *co-regulatory program structure*: genes that participate in shared or coupled regulatory cascades are encoded in related directions within the representation space, because the models learn to predict expression patterns shaped by regulatory relationships. This is consistent with the distributional hypothesis applied to transcriptomic data—genes that “co-occur” in similar expression contexts develop similar representations, and regulatory relationships generate specific co-occurrence patterns that leave geometric imprints.

This interpretation implies that geometric signal is a proxy for regulatory program membership, not a direct readout of TF binding. The signal should be strongest for regulatory relationships that produce clear, consistent expression signatures, and weakest for context-specific or transient interactions. This prediction is consistent with our observation that the signal is strongest in CD8^+^ T cells, where transcriptional reprogramming is dramatic and well-characterized, and weakest in relatively quiescent epithelial populations.

### The distributed nature of regulatory information in transformers

Perhaps the most important methodological insight is that regulatory information in transformer residual streams is distributed across layers and varies stochastically across random cell samples. The marked improvement from single-layer to multi-layer bundled scGPT representations—from near-null to a clear positive signal in the external-lung domain—demonstrates that different layers encode qualitatively different aspects of regulatory geometry.

This finding parallels results in NLP, where syntactic information concentrates in early-to-middle layers and semantic information in later layers of BERT-style models [21]. It suggests that interpretability studies of biological transformers should routinely evaluate multi-layer representations rather than selecting single “best” layers, which risks missing information encoded at non-optimal depths.

### Complementarity between model architectures

The partial complementarity between scGPT and Geneformer signals has both scientific and practical implications. The moderate edge-level correlation ($\rho \approx 0.47$) quantifies this complementarity: enough overlap to suggest both models capture real biology, but enough divergence to enable genuine information gain through combination. The enrichment of specific TF families in high-disagreement regions (GATA1, KLF1 in immune; HNF4A in lung) suggests that disagreement is biologically structured, reflecting genuine differences in how each model encodes specific regulatory programs.

Practically, this complementarity means that future improvements in either architecture can contribute additive value to the combined system. The compact stacking framework provides a principled mechanism for integrating such improvements.

### Comparison with attention-based approaches

Our findings should be interpreted alongside recent work showing that attention patterns in single-cell transformers primarily capture co-expression rather than direct regulatory relationships [11]. Taken together, these results suggest that while attention weights reflect statistical co-expression, deeper residual-stream representations encode subtler regulatory structure. This distinction is consistent with the transformer architecture’s design: attention computes pairwise token interactions within a layer, while the residual stream accumulates information across all layers through additive skip connections.

### Implications for gene regulatory network inference

Our results support a concrete but bounded role for foundation model geometry in GRN inference pipelines. We tested this directly: adding the geometric signal on top of expression-based GRN inference (GENIE3-style tree importance and co-expression) improved AUROC by *+0.023* to *+0.085* across domains under edge-level cross-validation, with all bootstrap confidence intervals excluding zero (Table 10). Used as a ranking score, the geometry-augmented model enriched true edges 1.6–*2.1× * over prevalence among the top 50 candidates per domain. Three practical uses follow, each with the caveat that the signal operates within a known gene universe and not for de novo prediction among unseen genes:

1. **Edge re-ranking:** geometric scores measurably complement expression-based methods when re-ranking candidate regulatory edges.
2. **Hypothesis prioritization:** for experimental follow-up (e.g. CRISPRi screens), geometric scores provide an orthogonal evidence channel, with quantified top-*k* enrichment.
3. **Context-dependent assessment:** because foundation model representations are conditioned on cellular context, geometric features can capture tissue-specific regulatory structure.

Directionality, which symmetric similarity metrics cannot provide, can be partially recovered: asymmetric per-layer features predict edge orientation at AUROC 0.80–0.90, so a practical pipeline could combine a symmetric score for edge presence with an asymmetric score for orientation.

### Limitations

Several limitations should be considered. First, TRRUST provides literature-curated edges that are biased toward well-studied genes and may contain false positives and false negatives. Second, effect sizes are modest in absolute terms: geometry-augmented AUROC reaches only 0.60–0.69, so the signal is statistical enrichment rather than a stand-alone regulatory classifier. Third, and most importantly, the signal is bounded by the entities seen in training: it is robust to leave-TF-out and leave-target-out cross-validation but collapses to near-zero under leave-both-out evaluation, so it supports re-ranking of candidate edges within a known gene universe rather than de novo prediction among entirely unseen genes. Fourth, the analysis is retrospective, and biological causality is not established—geometric proximity correlates with curated regulatory relationships but does not demonstrate mechanistic understanding; the cell-type interpretation in particular is offered as a hypothesis. Fifth, although a Benjamini–Hochberg correction across the full grid of comparisons leaves the headline results significant, the study involves many tissue/layer/metric/model contrasts and the exploratory analyses should be read as hypothesis-generating. Sixth, the cross-model comparison is sensitive to extraction choices; we mitigated this with a matched per-layer extraction for both models, but tokenization and cell-sampling differences between scGPT and Geneformer remain. Seventh, computational cost is non-trivial, though extraction is a one-time cost per model and dataset.

## Conclusions

We have presented a systematic investigation of whether residual-stream geometry in single-cell foundation models encodes gene regulatory information. Through analysis spanning four tissue contexts, two foundation model architectures, multiple geometric metrics, comprehensive null controls, grouped and confirmatory cross-validation, and a comparison against expression-based GRN inference, we establish three principal findings.

First, residual-stream geometry carries incremental signal for retrospective regulatory edge prioritization beyond expression-level confounds; the effect survives multiplicity correction, harder negative-edge constructions, and leave-TF-out and leave-target-out cross-validation, but it is bounded—absolute AUROC is modest and the signal collapses under fully out-of-entity (leave-both-out) evaluation. Second, extraction of this signal is critically dependent on methodological choices—particularly centered-cosine similarity, multi-layer bundling, and seed ensembling—and initial negative results in some domains were largely methodological rather than biological; under a comparable per-layer extraction the two foundation models carry similar geometric signal, and the previously apparent architectural gap was largely a representation-construction artifact. Third, the geometric signal complements expression-based GRN inference and carries partial directional information through asymmetric features, supporting a concrete role as an orthogonal evidence channel in multi-evidence edge re-ranking.

These findings advance our understanding of what single-cell foundation models learn about gene regulation and provide a practical framework for leveraging that knowledge in GRN inference. As foundation models continue to grow in scale and sophistication, systematic geometric analysis of their representations offers a promising path toward more reliable computational predictions of gene regulatory architecture.

## Methods

### Data sources and preprocessing

**Single-cell expression data.** We used processed single-cell RNA-seq datasets from the Tabula Sapiens atlas [19], which provides comprehensive transcriptomic profiles across human tissues. Four tissue contexts were analyzed: **kidney** (used for initial signal detection), **immune** (a composite of immune cell types including B cells, CD4^+^ T cells, and CD8^+^ T cells), **lung** (containing alveolar type I, alveolar type II, and macrophage populations), and **external lung** (an independent lung cohort used for orthogonal validation).

**Regulatory ground truth.** As a reference set of regulatory interactions, we used the TRRUST v2 database [22], which curates experimentally validated TF–target relationships extracted from published literature. TRRUST edges are directed (TF *→ * target) and annotated with a mode of regulation (activation, repression, or unknown); we used the TF*→ *target direction but not the activation/repression sign. After mapping to each tissue’s gene universe, the positive (and total, positive plus negative) edge counts were: kidney 288 (1152), immune 735 (2940), lung 8195 (32,780), and external lung 7504 (30,016).

**Negative edge construction.** For each domain, we constructed matched negative edges by sampling random (source TF, target gene) pairs from the same source and target gene pools as the positive edges, with a 3:1 negative-to-positive ratio in every domain.

### Foundation models

**scGPT.** We used the pre-trained scGPT whole-human model [8], a 12-layer transformer with generative pre-training on over 33 million single-cell profiles. For each tissue context, we sampled 256 cells (default seed 42, with additional seeds 43 and 44 for replication) and extracted per-layer residual-stream activations using forward hooks. The token budget was set to 512 genes per cell. Per-gene embeddings at each layer *ℓ * were obtained by averaging the corresponding token’s residual-stream vector across sampled cells:

1 $$\begin{aligned} \mathbf{h}_g^{(\ell )} = \frac{1}{|C_g|} \sum _{c \in C_g} \mathbf{r}_{g,c}^{(\ell )}, \end{aligned}$$

where $\mathbf{r}_{g,c}^{(\ell )} \in \mathbb {R}^d$ is the residual-stream vector for gene *g* in cell *c* at layer *ℓ *, $C_g$ is the set of cells in which gene *g* appears in the token sequence, and *d* is the model’s hidden dimension.

**Geneformer.** We used Geneformer [9] with its pre-trained token embeddings. Gene embeddings were extracted from the model’s embedding layer, producing a single static vector per gene. Gene mapping between the Geneformer vocabulary and each tissue’s expressed genes yielded coverage rates of 41.4% (immune), 83.2% (lung), and 77.2% (external lung).

### Geometric feature extraction

For each candidate TF–target pair $(g_s, g_t)$, we computed pairwise geometric features from the gene embeddings. Let $\mathbf{h}_s$ and $\mathbf{h}_t$ denote the source and target gene vectors, and let $\bar{\mathbf{h}} = \frac{1}{|G|}\sum _{g \in G} \mathbf{h}_g$ denote the global mean across all gene vectors. The following metrics were evaluated:

2 $$\begin{aligned} \text {Cosine similarity:} \quad&s_{\cos }(g_s, g_t) = \frac{\mathbf{h}_s^\top \mathbf{h}_t}{\Vert \mathbf{h}_s\Vert \, \Vert \mathbf{h}_t\Vert }, \end{aligned}$$

3 $$\begin{aligned} \text {Centered cosine:} \quad&s_{\text {cc}}(g_s, g_t) = \frac{(\mathbf{h}_s - \bar{\mathbf{h}})^\top (\mathbf{h}_t - \bar{\mathbf{h}})}{\Vert \mathbf{h}_s - \bar{\mathbf{h}}\Vert \, \Vert \mathbf{h}_t - \bar{\mathbf{h}}\Vert }, \end{aligned}$$

4 $$\begin{aligned} \text {Negative } L_2 \text { distance:} \quad&s_{L_2}(g_s, g_t) = -\Vert \mathbf{h}_s - \mathbf{h}_t\Vert _2, \end{aligned}$$

5 $$\begin{aligned} \text {Dot product:} \quad&s_{\text {dot}}(g_s, g_t) = \mathbf{h}_s^\top \mathbf{h}_t. \end{aligned}$$

**Low-rank variants.** For PCA-projected features, we computed the top-*k* principal components of the gene embedding matrix $\mathbf{H} \in \mathbb {R}^{|G| \times d}$ for $k \in \{32, 64, 128\}$, projected all gene vectors into this subspace, and computed pairwise metrics in the reduced space.

**Multi-layer bundling.** We concatenated geometric features across multiple scGPT layers rather than selecting a single best layer. For a layer bundle $\mathcal {L} = \{0, 1, \ldots , L\}$, the feature vector for a candidate edge becomes $\mathbf{f}_{g_s, g_t} = [s^{(\ell )}(g_s, g_t)]_{\ell \in \mathcal {L}}$.

**Seed ensembling.** We averaged geometric features across multiple random seeds (42, 43, 44) before downstream classification: $\bar{s}(g_s, g_t) = \frac{1}{3}\sum _{i} s^{(\text {seed}_i)}(g_s, g_t)$.

### Edge classification framework

For each tissue domain, we formulated a binary classification task: given a candidate TF–target pair, predict whether it is a curated TRRUST regulatory edge (positive) or a matched non-regulatory pair (negative).

**Baseline features.** All models included baseline confound features: mean expression, variance, and detection frequency for both source and target genes (six features total).

**Model comparison.** For each domain, we compared: (1) **Baseline-only**: logistic regression on six confound features; (2) **Baseline + geometry**: logistic regression adding geometric features to the baseline. The primary metric is ΔAUROC: the AUROC difference between the geometry-augmented and baseline-only models; we additionally report absolute AUROC, absolute AUPRC, ΔAUPRC and precision/recall at fixed cut-offs.

**Implementation detail.** All classifiers are scikit-learn pipelines consisting of StandardScaler followed by LogisticRegression (L2 penalty, lbfgs solver, class_weight=balanced, max_iter=2000); features are standardized within each training fold only. PCA-projected features use randomized SVD on the globally centered per-layer gene-embedding matrix, retaining $k \in \{32, 64, 128\}$ components fitted within training data. For scGPT, 256 cells were sampled per domain (seeds 42/43/44) with a 512-gene token budget; per-gene layer embeddings are the mean of the token’s residual-stream vector over cells in which the gene appears. Gene mapping uses each tissue’s expressed-gene universe; edges are retained only when both genes have non-zero embedding coverage and finite confound features.

### Evaluation protocol

Edge-level predictive evaluations used repeated stratified 5-fold cross-validation (3 repeats); grouped evaluations used the leave-TF / leave-target / leave-both-out splits described below. For each ΔAUROC estimate, 95% bootstrap confidence intervals were computed from at least 400 resamples (1000 for the headline grouped, confirmatory and cross-model analyses). For the compact stacking model, we used nested cross-validation: in the outer loop, data is split into train and test folds; within each outer fold, branch models are trained using inner cross-validation, and out-of-fold predictions serve as features for the meta-learner.

### Null controls

**Label permutation.** We randomly permuted edge labels while preserving feature structure, then re-estimated ΔAUROC under permuted labels (*n = 40* repeats). The empirical *p*-value is the fraction of permutation values exceeding the observed ΔAUROC.

**Geometry-feature shuffle.** We randomly permuted geometric scores across edges while preserving baseline features and labels (*n = 60* repeats). This null tests whether the geometric feature identity, not merely the presence of an additional feature, drives the observed improvement.

### Compact stacking model

The compact combiner integrates geometric signals from both foundation models through a stacked architecture: (1) train branch model A (baseline + scGPT) and B (baseline + Geneformer); (2) generate inner-fold OOF probability predictions; (3) train meta-model on baseline features + OOF predictions; (4) evaluate on held-out outer folds.

### Grouped, leakage-resistant cross-validation

To test out-of-entity generalization beyond edge-level random splits, we used three grouped cross-validation schemes. *Leave-TF-out* and *leave-target-out* use GroupKFold (*k=5*) keyed by the source TF or the target gene respectively, so that no TF (respectively target) appears in both training and test folds. *Leave-both-out* partitions TFs and targets independently into *b=4* random blocks; for block *k* the test fold is the set of edges whose TF and target both fall in block *k*, and the training fold is the set of edges whose TF and target both fall outside block *k*; crossover edges are excluded from that fold. For grouped schemes, bootstrap resampling is performed at the level of gene groups (cluster bootstrap) rather than individual edges.

### Negative-edge sampling and robustness

The primary negative set is sampled once by uniform rejection sampling of (source-pool TF, target-pool gene) pairs excluding positives, at a 3:1 negative-to-positive ratio in every domain. For the robustness analysis we additionally generated negatives under two harder protocols at a balanced 1:1 ratio (chosen so that the harder-negative comparison is not confounded by class ratio): *degree-matched*, in which each negative’s TF and target are drawn from the same TRRUST out-degree / in-degree quantile bin (5 bins) as the corresponding positive; and *expression-matched*, matched instead on mean-expression and detection-frequency bins. Each protocol was repeated for eight independent random draws, and we report the mean and standard deviation of ΔAUROC, AUROC and AUPRC across draws.

### Uncertainty decomposition and multiplicity

We decomposed the variance of ΔAUROC into a within-run component (from the edge or cluster bootstrap) and an across-seed component (the standard deviation of the point estimate across cell-sampling seeds 42/43/44, which also varies the negative draw). Reported confidence intervals are widened to incorporate the across-seed term. Across the full grid of domain *× * layer *× * metric comparisons, one-sided *p*-values (normal approximation from the bootstrap interval) were corrected for multiplicity with the Benjamini–Hochberg procedure at *q < 0.05*.

### Exploratory versus confirmatory analyses

Representation choices (geometric metric, PCA dimension, layer bundle, seed-aggregation strategy) were selected during exploratory analysis on the kidney and immune domains. For the confirmatory evaluation we froze a single pre-specified pipeline—centered-cosine similarity, multi-layer bundle L0–11, logistic-regression stacking, with no per-domain tuning—and applied it once to the held-out confirmatory domains (lung and external lung) across three seeds.

### Geneformer residual-stream extraction

For the matched cross-model comparison we extracted Geneformer residual-stream activations analogously to scGPT. We used the pre-trained Geneformer model (ctheodoris/Geneformer; 18-layer transformer, hidden dimension 1,152) and sampled 192 cells per domain. Each sampled cell was tokenized with the official rank-value encoding: counts were normalized to a fixed total, divided by corpus gene medians, and ranked; the top 2,048 genes were wrapped in the model’s $\texttt {<cls>}/\texttt {<eos>}$ tokens. Per-layer hidden states were captured for all 18 layers and averaged across cells into per-gene embeddings, after which the identical geometric feature pipeline was applied to both models.

### Directionality analysis

Because the geometric similarity metrics are symmetric in (source, target), we tested directionality with antisymmetric per-layer features—signed differences in vector norm, squared norm, and mean coordinate between the two genes—whose sign flips when the pair is reversed. For each curated edge we created a correctly oriented example (label 1) and its reverse (label 0) and trained a logistic-regression classifier under GroupKFold keyed by the unordered pair, so that a pair and its reverse never separate across folds. A complementary gene-level task classified TF genes versus target-only genes from PCA-reduced embeddings.

### Ensembling with expression-based GRN inference

To assess incremental value over established GRN inference, we computed two expression-based edge scores on each domain’s single-cell matrix restricted to the TRRUST gene universe: the absolute Pearson correlation between TF and target expression (co-expression), and a GENIE3-style [4] score in which a random-forest regressor (40 trees, maximum depth 8) predicts each target gene’s expression from all candidate TFs and the TF*→ *target feature importance is the edge score. We then compared a confound + GRN logistic-regression model against a confound + GRN + geometry model under edge-level and leave-both-out cross-validation.

## Data Availability

The Tabula Sapiens atlas is available at https://tabula-sapiens-portal.ds.czbiohub.org/. The TRRUST v2 database is available at https://www.grnpedia.org/trrust/. The scGPT pre-trained model is available at https://github.com/bowang-lab/scGPT. The Geneformer pretrained model is available on Hugging Face at https://huggingface.co/ctheodoris/Geneformer. All analysis code, environment specifications, and reproduction instructions are available at https://github.com/Biodyn-AI/geometric-residual-stream.
